# Food and Drug Administration novel drug decisions in 2017: transparency and disclosure prior to and 5 years following approval

**DOI:** 10.1093/haschl/qxad028

**Published:** 2023-07-19

**Authors:** Robert M Kaplan, Amanda J Koong, Veronica Irvin

**Affiliations:** Clinical Excellence Research Center, Stanford University School of Medicine, Stanford, CA 94305, United States; University of Texas Health Science Center at Houston, McGovern School of Medicine, Houston, TX 77030, United States; College of Health, Oregon State University, Corvallis, OR 97331, United States

**Keywords:** FDA, regulatory science, evidence-based medicine, evidence standards

## Abstract

The Food and Drug Administration (FDA) approved 46 novel drugs in 2017. We reviewed availability of results prior to and during the 5 years following each approval. Using the FDA website and ClinicalTrials.gov, we recorded trials cited as evidence for the approval, total number of studies registered in ClinicalTrials.gov, number started and completed before approval, and the frequency and timing of reporting results. The 46 drugs approved in 2017 were evaluated in 1149 studies. The number of studies used to evaluate the 46 drugs ranged from 2 to 165 (mean: 24.98; SD = 28.95). Among these, an average of 9.22 studies (SD = 9.21) were started and 5.82 studies (SD = 6.89) were completed before the approval. A single trial justified approval for 19 of 46 (41%) of the approved products. Public posting of results prior to the FDA approval was available for an average of only 1.42 studies (SD = 3.12). No results were publicly reported before approval for 9 of the 46 drugs (20%). Health care providers and consumers depend on complete and transparent reporting of information about FDA-approved medications. Only a fraction of evidence from completed studies was available before approval and a substantial portion of research evidence remained undisclosed after 5 years.

## Introduction

Although US Food and Drug Administration (FDA) approval is assumed to assure medication safety and efficacy, there has been an increasing number of challenges to the evidence base supporting some recent approval decisions.^1–3^

In December of 2016, President Obama signed the 21st Century Cures Act. The Act, which had wide bipartisan support, was designed to accelerate the approval of new medical products and allow patients earlier access to new innovations. To achieve these goals, the Act relaxed some FDA standards by allowing treatments for priority health conditions to be approved with fewer supporting studies. In addition, it signaled greater flexibility for using surrogate markers rather than clinical outcomes, and it placed less emphasis on traditional research methods such as the randomized clinical trial.^3^

Current FDA policy requires pharmaceutical companies to submit 2 completed trials with their approval request.^4^ For high-priority conditions, such as some cancers, approvals can be based on a single trial. However, companies are allowed to choose which trials to submit and do not have to disclose additional studies that might have shown less or nonsignificant effectiveness. Since the enactment of the Act, the number of approved new medicines has increased. Between 2006 and 2016, an average of 29 new products were approved each year. In 2017 when the Cures Act in 2017 was implemented, approvals jumped to 46 and then to 59 in 2018.

In this report, we examine all new approvals for the calendar year 2017. We chose 2017 because it was the first year following the passage of the 21st Century Cures Act. In addition, the FDA regulation requiring posting of results on ClinicalTrials.gov took effect in January of 2017. Further, focusing on 2017 allows an evaluation of what happened in the 5 years following product approval. Specifically, the purpose of this study is to document how many studies were initiated, completed, and reported before and after the approval.

## Methods

### Sample frame and data

Using the FDA website, we examined all novel drug approvals for the 2017 calendar year. For each approved medication, we used the National Library of Medicine ClinicalTrials.gov website to identify when each study was first posted, the start date, completion date, and when or whether results were publicly reported. For each study, all of the standardized fields in ClinicalTrials.gov were downloaded into a comprehensive spreadsheet.

### Completed trials

For each new approval listed in the FDA report, we coded the number of trials used for the approval, the total number of studies registered, the number of completed studies, and the studies reporting results in ClinicalTrials.gov. Similarly, we recorded whether results were posted on ClinicalTrials.gov prior to or after the FDA approval. For studies that reported results, we noted whether the results were posted shortly after the approval, defined as within 9 months, or if they had been reported at all within 5 years. The end date for defining this period was December 2022.

## Results

The 46 drugs approved in 2017 have been evaluated in 1149 studies. Table 1 summarizes the number of trials used for the approval, the total trials initiated before approval, the number initiated after approval, the number of studies completed before the approval, the number reporting results before the approval, and the total number reporting results within 5 years of FDA approval.

**Table 1.** Summary of trials used for 46 drugs approval by the FDA in 2017 and those initiated, completed, and reported prior to approval.

Summary of studies	Mean	SD
Number of trials in FDA approval^a^	2.2	1.71
Total trials registered on ClinicalTrials.gov^b^	24.67	29.53
Started before FDA approval^c^	9.22	9.21
Completed before FDA approval^d^	5.82	6.89
Results posted before FDA approval^e^	1.42	3.12
Results ever posted^f^	8.38	8.39
**Frequencies and timing**	**Percentage**	
Started before FDA approval	47%	
Completed before FDA approval	49%	
Results posted before FDA approval	6%	
Results ever posted	34%	
Posted at least 1 new result within 9 months after approval	72%	
Multiple results posted within 9 months after approval	43%	

Abbreviation: FDA, Food and Drug Administration.

a Derived from Drug Trial Snapshots or press releases cited on the FDA website (https://www.fda.gov/drugs/new-drugs-fda-cders-new-molecular-entities-and-new-therapeutic-biological-products/novel-drug-approvals-2017).

b Based on searches in ClinicalTrials.gov by active ingredient and indication.

c Based on start date from ClinicalTrials.gov and approval date listed on FDA website.

d Based on completion date from ClinicalTrials.gov and approval date listed on FDA website.

e Based on results posting date from ClinicalTrials.gov and approval date listed on FDA website.

f Mean number of studies reporting result in ClinicalTrials.gov across 46 products.

In 2017, 19 of 46 (41%) new approvals were based on a single study. On average, the approvals were based on 2.2 studies (SD = 1.71). The number of studies in ClinicalTrials.gov (as of December 2022) used to evaluate the 46 drugs approved in 2017 ranged from 2 (for Vavomere, Xepi, and Macrilen) to 165 (Ozempic, Novo Nordisk) (mean: 24.98; SD = 28.95). Among these, an average of 9.22 studies (SD = 9.21) were started before the approval and 5.82 studies (SD = 6.89) were completed before the approval. Yet, on average, only 1.42 studies (SD = 3.12) had disclosed results prior to the approval. Among the products that ever disclosed results, less than half of the relevant studies posted results in ClinicalTrials.gov within 5 years.

For studies posting results after the approval, we examined when the posting occurred. For 33 of the 46 approved medications (72%), at least 1 new result was first posted on ClinicalTrials.gov within 9 months after approval, and for 20 of the 46 (43%) drugs, more than 1 study posted results within 9 months of approval. For 4 products, 100% of publicly available results were first disclosed after FDA approval. Five years after approval, results had yet to be disclosed on ClinicalTrials.gov for 13 of 46 products (28%) approved by the FDA in 2017.

## Discussion

Our findings suggest that important results relevant to newly approved pharmaceutical products are often not reported as required by the FDA Amendments Act (FDAAA).^5^ The FDAAA includes specific policies to address nonpublication of results from clinical trials.^6^ Zarin and colleagues^7^ similarly reported that only about half of completed trials reported results in ClinicalTrials.gov. This is higher than our observation of 41% for 2017. Over the course of time, the gap between completed trials and trials reporting results appears to be widening. Piller^8^ described an investigation by *Science News* that considered over 4768 studies that should have posted results on ClinicalTrials.gov. They found that about 32% (1506 studies) failed to report the results and that less than half (44.7%) reported their results on time.

Zhang and colleagues^9^ examined 273 new drug approvals in 3 time periods: 1995–1997, 2005–2007, and 2015–2017. Consistent with our observations, they found that more recent approvals were based on fewer pivotal trials and trials that used less rigorous research designs. Darrow and colleagues^1^ reviewed FDA databases for new approvals between 1984 and 2018. Consistent with our results, they found substantial upticks in new approvals over the last few years.

We are unaware of other investigations of studies that were reported shortly after a product achieved approval. We observed this practice to be common, occurring among 72% of drugs approved in 2017. Frequently, these studies had been completed several years before the approval date. Although we do not know why these studies were not reported within the required 1-year interval following completion, we speculate that data were not posted because they did not clearly support the case for approval. If this speculation is correct, it would imply that clinical trials used for FDA approval may be a highly selected subset of completed research studies.

The results of the study should be interpreted while considering several limitations. Our focus is on the posting of results in ClinicalTrials.gov, not on the FDA process. It is possible that the FDA received results from clinical trials prior to their posting on ClinicalTrials.gov. If that is true, our assumption that the FDA is not reviewing all information may be inaccurate. However, the FDA has the responsibility to disclose information that is relevant to consumer and provider decisions to use medications. It seems unlikely that the FDA would deliberately withhold information from their advisory boards and the public. We also recognize that study results may have been disclosed at professional meetings or in publications even though they were not posted in ClinicalTrials.gov, as required by the FDAAA.

A second concern is that we do not know for certain what information the FDA uses in their decision process. Although advisory committee meetings are open to the public, the FDA is not obliged to follow committee recommendations. However, the FDA states specifically which studies were used to justify each approval. This information is shown in “Drug Trial Snapshots” or in FDA press releases and our analysis reviewed both sources. We recognize that applicants may cite other studies in presentations to advisory committees and in literature reviews. Further, decision-makers may be influenced by information other than studies formally identified in the approval.

A final limitation is that the 9-month window for recording whether results were reported shortly after approval was chosen somewhat arbitrarily. We felt that 6 months was too short because, following a new approval, key staff are often diverted to the product launch. On the other hand, a 1-year interval may be too long. The 9-month interval fell in-between. We recognize that the use of a different cut point might affect the number of studies identified as reporting results shortly after approval.

In summary, drugs approved in 2017 appeared to be based on a subsample of information on product safety and efficacy. Only about one-quarter of completed studies made results public before approval. Further, it is not uncommon for results to be reported shortly after approval, suggesting that companies might release or hold results to show their products in the best light. Despite efforts by the FDA to increase transparency, investigators are still able to selectively report outcomes for variables that favor treatment effectiveness. One recent report noted that 10% of approvals were granted despite null findings for the primary outcome variable. Only about half (48%) were approved on the basis of clinical measures and about one-quarter (26%) were approved on the basis of surrogate markers.^10^ Among the drugs that gained FDA approval between 2017 and 2020, 5 had been refused approval by FDA peer agencies in other countries. Further, health technology agencies in Australia, Canada, or the United Kingdom had declined to endorse 42 of the products because the benefits were uncertain or the costs were excessive.^11^ Prescribers, purchasers, and patients depend on transparent and rigorous review of pharmaceutical products. Our evaluation suggests we can do better.

## Supplementary Material

qxad028_Supplementary_Data
